# Microalgal kinetics — a guideline for photobioreactor design and process development

**DOI:** 10.1002/elsc.201900107

**Published:** 2019-10-14

**Authors:** Kira Schediwy, Andreas Trautmann, Christian Steinweg, Clemens Posten

**Affiliations:** ^1^ Institute of Process Engineering in Life Sciences, Section III: Bioprocess Engineering Karlsruhe Institute of Technology (KIT) Karlsruhe Germany; ^2^ Process Scale Up and PD Support, Lonza AG Visp Switzerland

**Keywords:** carbon uptake kinetics, growth integration, light response curve, macromolecular composition, microalgae

## Abstract

Kinetics generally describes bio‐(chemical) reaction rates in dependence on substrate concentrations. Kinetics for microalgae is often adapted from heterotrophs and lacks mechanistic foundation, e.g. for light harvesting. Using and understanding kinetic equations as the representation of intracellular mechanisms is essential for reasonable comparisons and simulations of growth behavior. Summarizing growth kinetics in one equation does not yield reliable models. Piecewise linear or rational functions may mimic photosynthesis irradiance response curves, but fail to represent the mechanisms. Our modeling approach for photoautotrophic growth comprises physical and kinetic modules with mechanistic foundation extracted from the literature. Splitting the light submodel into the modules for light distribution, light absorption, and photosynthetic sugar production with independent parameters allows the transfer of kinetics between different reactor designs. The consecutive anabolism depends among others on nutrient concentrations. The nutrient uptake kinetics largely impacts carbon partitioning in the reviewed stoichiometry range of cellular constituents. Consecutive metabolic steps mask each other and demand a maximum value understandable as the minimum principle of growth. These fundamental modules need to be clearly distinguished, but may be modified or extended based on process conditions and progress in research. First, discussion of kinetics helps to understand the physiological situation, for which ranges of parameter values are given. Second, kinetics should be used for photobioreactor design, but also for gassing and nutrient optimization. Numerous examples are given for both aspects. Finally, measuring kinetics more comprehensively and precisely will help in improved process development.

**Abbreviations**
CCM: carbon concentration mechanisms
Chl: chlorophyll
CTR: carbon dioxide transfer rate
PI‐curve: photosynthesis irradiance response curve
PSU: photosynthetic units
RuBisCo: ribulose‐1,5‐bisphosphate carboxylase/oxygenase

## 1 INTRODUCTION

Photosynthesis is the major biochemical process to drive life on earth. Heterotrophic life only functions by respiration of oxygen supplied by phototrophs. Microalgae — next to terrestrial plants — contribute substantially to the oxygen evolution. The amount of oxygen produced by microalgae is remarkable due to their high photosynthetic efficiency. Efficient light use gives microalgae great potential for applications in pharmaceutical, cosmetic, food, feed, and chemical industries 1. Designing microalgal production plants is an emerging field developing more and more into the direction of rational process design.

For heterotrophic bio‐processes, the rational basis of process design is well elaborated. For photo‐bioprocesses, mechanistic knowledge is described in the literature, but not straightforwardly applied on process development. For process development, production plants can be hierarchically structured into a plant/reactor level, the level of the microalgal population (suspension), and the level of the intracellular metabolic network 2. Kinetics on the population level links the reactor and the cell level. To represent valid connections the kinetics has to be as rational as possible. The structure of each kinetic equation should reflect the structure of the real system. Unknown kinetic parameters should at least have a clear physiological meaning. Further, the parameters have to be independent from scale 2.

Lee et al. 3 and Béchet et al. 4 recently reviewed kinetic models for microalgal growth. A short summary of different mathematical descriptions including examples is given in Table 1. Next to classical kinetic models of Monod 5, Blackman 6, and Andrews 7, new expressions have been proposed in the last decades especially for light kinetics 8. Classical kinetics is based on mass action law and reaction kinetics, and uses enzyme kinetics as template. Both do not consider the physical step of light absorbance depending on chlorophyll content in the chloroplast. Other mathematical attempts are empiric or semi‐mechanistic 9. Light limitation and light inhibition are completely different processes on different time scales, but are formulated with only one term in the kinetics, e.g. Steele 10. This can be overcome with kinetics defined piecewise, where the classic piecewise linear photosynthesis irradiance response curve (PI‐curve) is one example. Most measured PI‐curves for adapted cultures in photo‐bioreactors exhibit a distinct saturation range before reaching inhibition. The broad range of saturation may be due to a limiting step downstream from sugar production in the metabolism, which is considered by Blackman kinetics 6. The model according to Han is one of the few being directly derived from reaction kinetics of photosynthetic units 8. This will be derived later in this review (see Section 2.1).

**Table 1:** Overview of the structure of commonly used kinetics

Mathematical approach	Examples	Advantages	Disadvantages	References
Rational functions (different exponents in numerator and denominator)	Andrews, Monod, Haldane	Based on reaction or enzyme kinetics	No specific consideration of phototrophic light processing	22, 75, 76
Rational function with mechanistic meaning in photosynthesis (formally as Monod)	Han	Based on chlorophyll reaction kinetics	PSU and time constants not directly measurable	17, 19, 77, 78 [see also this review]
Special functions (exponential, hyperbolic tangent)	Steele (exp), Jassby & Platt (tanh), van Oorschot (exp)	Easy to use with some flexibility	No claim to mechanistic background, if then thermodynamics	10, 14, 79
Piecewise defined functions	Classical PI‐curve, Blackman	Clear discrimination between dominant processes over light range	Numerical point of discontinuity, over‐simplification in transition zones	24, 80 [see also this review]
Multiplication of different kinetic factors/equations	Commonly applied to combination of different influences	Simple to use even in complex environment	Ignores intracellular stoichiometry	56

Several aspects impede finding and validating kinetics for photo‐bioprocesses. The first one is the temporal aspect. Photo‐acclimation ranges from fractions of a second to hours or days 11, 12. The differences in time scales prohibit the transfer of short‐term measurements to outdoor cultivations. Another aspect is the spatial characteristic of light gradients. The gradients make the direct application of kinetics possible for only one light intensity. The necessary light integration over the reactor volume and the consequences for the appearance of the kinetics will be discussed later in this review. The third aspect is the variability of the cells. Cellular composition varies according to heterotrophic kinetics depending on nutrients in the medium. Substrates for heterotrophic pathways are carbohydrates formed in the chloroplast. The chloroplast performs light absorption and photosynthesis partially independent from the heterotrophic pathways of the cell. This variability of cellular reactions, time constants of reactions, and acclimation as well as light gradients are indispensable for the simulation of microalgal physiology.

Only mechanistic models based on physiological understanding of the cell yield reliable predictions when applied to other process conditions later on. Well formulated and reasonably simplified kinetics can then be coupled to hydrodynamics and light attenuation in one model 13. Such models help to analyze and optimize cultivation systems or to design new reactors and microalgae production plants. We will show that only one kinetic equation for growth as a function of light is not enough to consistently represent the cell's behavior. Building up on reactor conditions and intracellular stoichiometry, separate equations for photon absorbance and growth lead to a consistent and scale independent system of kinetics. The necessary biological knowledge for the kinetic equations is in many cases already available or can be retrieved in small scale experiments. Based on this kinetics, we will show how powerful such an approach can be for reactor and medium design as well as process development in general. The presented approach is not comprehensive for the diversity of technical and biological situations, but will encourage going further into the direction of more knowledge driven rational process development for photo‐bioprocesses.

## 2 LIGHT KINETICS — A SCAFFOLD TO OPTIMIZE PHOTOBIOREACTOR GEOMETRY

Setting up kinetics for phototrophic bioprocesses can be carried out in analogy to heterotrophic bioprocesses. For heterotrophs substrate uptake is usually assumed to be an enzymatic step. The specific substrate uptake rate *r*_S_ = *f*(*c*_S_) [g·(g·h)^−1^] is represented by Michaelis–Menten‐type kinetics. The substrate uptake can be summarized as rational function kinetics, with a polynomial expression in numerator and denominator, considering also different kinds of inhibition. In growth kinetics, a stoichiometric equation for the specific growth rate *r*_X _= *f*(*r*_S_) [g·(g·h)^−1^] as function of substrate uptake is formulated (Equation 1).

(1) $$\begin{array}{c} \begin{array}{cc} r_{S}\left(c_{S}\right)=r_{S,\max}\cdot \frac{c_{S}}{c_{S}+k_{S}} & \mathrm{substrate}\;\mathrm{uptake}\\ r_{X}\left(r_{S}\right)=y_{X,S}\cdot r_{S}-r_{X,m} & \mathrm{substrate}\;\mathrm{usage} \end{array} \end{array}$$

The yield coefficient *y*_X,S_ [g·g^−1^] and the maintenance parameter *r*_X,m_ [h^−1^] are interpreted from assumptions about carbon and energy balance. From the combination of substrate uptake and yield the Monod equation *r*_X_ = f(c_S_) can be deduced. Splitting up Monod kinetics into substrate uptake and yield is in many respects no true mechanism, but at least follows one clear cause‐effect chain. This practicability has given reason to adapt Monod‐type kinetics from heterotrophic growth 4 or other formal kinetics to phototrophs 10, 14. In the phototrophic case, things are different. First, light is not a concentration but a flux. Reactor equations for light compare to equations describing a fed‐batch with linear feeding. Second, light uptake is not enzymatic, but a linear physical absorbance step. Growth may be stoichiometrically coupled to absorbed photons in analogy to the heterotrophic yield equation.

### 2.1 The photosynthetic response curve — the basic building block of microalgal growth kinetics

Setting up light kinetics as a quantitative way to describe the cellular response to irradiance has been regarded as an important concern for decades 15. Measuring the photosynthetic activity as a function of light intensity in the photosynthetically active frequency range leads to the so‐called PI‐curve. This curve can be given either as a function of the (local) photon flux density here denoted as *I*_hν_ [µmol·m^−2^·s^−1^] or as the function of the specific absorbed photon flux *r*_hν,abs_ [µmol·g^−1^·s^−1^]. Absorbed photons are potentially active in photosynthesis and, thus describing kinetics based on absorbed photons allows for a better understanding of the underlying physiological effects. In the following paragraphs three different kinetic approaches building upon each other will be reviewed and discussed especially for the specific growth rate *r*_X_ [g·g^−1^·h^−1^] as the photosynthetic activity.

In the simplest case, the specific growth rate as function of irradiance starts with a linear increase for low light intensities. The slope *y*_X,I_ = dr_X_/dI_hν_ is a measure for the sensitivity of growth to light. The negative intercept of the specific growth rate *r*_X_ accounts for maintenance energy *r*_X,m_. On the first linear increase follows a more or less constant course of *r*_X_ at medium light intensities. A limiting step in the metabolism leads to a maximum specific growth rate *r*_X,max_ under the given environmental conditions. Possibly, at high irradiance values, light inhibition causes a decreasing part of the curve. As light inhibition is a multi‐factorial process on different time scales and should be avoided during production, it is not further discussed in this review. The specific growth rate *r*_X_ can now be formally represented as Equation 2.

(2) $$\begin{array}{ccc} & & r_{X}\left(I_{h\nu }\right)\\ & & \;=\left\{\begin{array}{cc} y_{X,I}\cdot I_{h\nu }-r_{X,m} & \mathrm{for}\;\;\;I_{h\nu }<I_{h\nu ,sat}\\ r_{X,\max} & \mathrm{for}\;\;\;I_{h\nu ,sat}<I_{h\nu }<I_{h\nu ,inhi} \end{array}\right. \end{array}$$

This kinetic approach contains three a‐priori unknown physiological parameters being *r*_X,max,_ *r*_X,m_, and *y*_µ,I_. The specific light intensities compensation point *I*_hν,comp_ for *r*_X_ = 0, the onset of saturation *I*_hν,sat_ and the onset of inhibition *I*_hν,inhi_ can be deduced from the physiological parameters as given in Equation 2 or vice versa. This simple kinetics already allows to solve different tasks in reactor design as outlined in the last paragraph of this section.

Kinetics does not develop full expressiveness until being matched against underlying physiological mechanisms. The first step of interaction between light intensity and growth is light absorption, a linear process. Absorption is determined by the effective absorption cross section σ_X_ [m^2^·g^−1^] of the biomass. The specific absorbed photon flux *r*_hν,abs_ [µmol·g^−1^·s^−1^] is a measure for the potentially available photosynthetic energy of the cell under light limiting conditions. The energy uptake by physical absorption justifies the linearly increasing part of the kinetics under light limitation. Light saturation under optimal growth conditions can depend on (unknown) intracellular bottle‐necks *r*_int_ in photosynthesis. The excess energy of absorbed photons is then dissipated as fluorescence irradiation or heat, so called non‐photochemical quenching 16. Equation 3 gives the kinetic approach based on absorbed photons 4.

(3) $$\begin{array}{c} \begin{array}{cc} r_{h\nu ,abs}=\sigma_{X}\cdot I_{h\nu } & \mathrm{light}\;\mathrm{absorption}\\ r_{X}\left(r_{h\nu ,abs}\right)=y_{X,h\nu }\cdot r_{h\nu ,abs}-r_{X,m} & \mathrm{light}\;\mathrm{limitation}\\ r_{X}\leq r_{X,\max}\; & \mathrm{light}\;\mathrm{saturation} \end{array} \end{array}$$

In contrast to substrate uptake in the heterotrophic case (Equation 1), absorption as such has no natural limit, so in this kinetic model the maximum specific growth rate is determined by an intracellular step. The yield parameter *y*_X,hν_ [g·mol^−1^] has a clear physiological meaning being the photosynthetic efficiency of formed biomass on absorbed photons.

Many authors observed no linear increase but a smooth saturation curve without showing a sharp kink in the transition between light limitation and light saturation. The reason of this non‐linear behavior can be understood as partial saturation of the light harvesting complexes. Chlorophyll (Chl) molecules change between an excited state after being hit by a photon and a reactive state after the energy has been transferred to the active center. The time constant τ_Chl_ [s] related with this energy transfer process (mostly assumed for PSII) is decisive for the total photosynthetic rate. Excited Chl molecules can also fall back to the reactive state. The excess energy is then lost by heat and fluorescence, reducing photosynthetic efficiency even at moderate light intensities. Chlorophyll molecules, which are already in the excited state, cannot be further excited by a second photon.

A widely accepted kinetic model based on these assumptions has been given by Han 8 and further elaborated by Bernardi 17, 18. Their state model is based on photosynthetic units (PSU). Each PSU consists of Chl molecules and the following steps in PSII, PSI, and carbon fixation to form one molecule of oxygen. PSUs can be reactive or activated (open, closed). However, it is not clear, what these states mean physically for PSU. In the following equations the model is adapted to biomass as the system boundary to contain only macroscopically measurable parameters. The first step considers light absorption as in Equation (3). But only Chl molecules being in the reactive state can change their state into excited, assuming that exactly one photon per Chl is involved. The specific number of reactive Chl is here denoted as *n*_Chl,reactive_ and the Chl in the excited state as *n*_Chl,excited_ [‐], where n_Chl_ is the number of Chl molecules per biomass [1·g^−1^]. Excited Chl can then transfer energy to the reaction center via first order reaction, falling back to the reactive state. The physiological interpretation of the time constant τ_Chl_ [s] could be the relaxation time of PSII but is not clearly described in references. The concept of first order kinetics does not actually require an explicit time constant in a following step after light capture. Setting up mass balances leads to differential equations for each of the two states and an algebric equation for the whole Chl as given in Equation (4).

(4) $$\begin{array}{c} \begin{array}{c} \frac{dn_{Chl,reactive}}{dt}=-\sigma_{X}\cdot I_{h\nu }\cdot \frac{n_{Chl,reactive}}{n_{Chl}}+\frac{n_{Chl,excited}}{\tau_{Chl}}=0\\ \frac{dn_{Chl,exicted}}{dt}=+\sigma_{X}\cdot I_{h\nu }\cdot \frac{n_{Chl,reactive}}{n_{Chl}}-\frac{n_{Chl,excited}}{\tau_{Chl}}=0\\ n_{Chl,reactive}+n_{Chl,excited}=n_{Chl} \end{array} \end{array}$$

With respect to time constants of the growth process being orders of magnitudes higher than τ_Chl_, these two linearly dependent differential equations can be considered to be stationary (d/dt = 0). This assumption leads to the specific transport rate *r*_hν,act_ [mol·g^−1^·s^−1^] of excited photon energy (excitons) finally being active in water splitting:

(5) $$\begin{array}{ccc} r_{h\nu ,act} & = & \frac{n_{Chl,excited}}{\tau_{Chl}}\\ & = & \frac{\sigma_{X}\cdot I_{h\nu }\cdot n_{Chl}}{\sigma_{X}\cdot I_{h\nu }\cdot \tau_{Chl}+n_{Chl}}\\ & = & \frac{n_{Chl}}{\tau_{Chl}}\cdot \frac{I_{h\nu }}{I_{h\nu }+\frac{n_{Chl}}{\sigma_{X}\cdot \tau_{Chl}}} \end{array}$$

Unknown parameters can be lumped to achieve a workable kinetics with less and measurable parameters:

(6) $$\begin{array}{ccc} r_{h\nu ,act}\left(I_{h\nu }\right) & = & r_{h\nu ,act,\max}\cdot \frac{I_{h\nu }}{I_{h\nu }+k_{I}}\;\;\;\;\;\mathrm{with}\;\;r_{h\nu ,act,\max}\\ & = & \frac{n_{Chl}}{\tau_{Chl}}\;\;\;\;and\;\;\;k_{I}=\;\frac{n_{Chl}}{\sigma_{X}\cdot \tau_{Chl}} \end{array}$$

To finally obtain a growth kinetics, the yield of biomass per photon *y*_X,hν_ [g·mol^−1^] is included as well as a maintenance parameter *r*_X,m_ [h^−1^] describing the energy demand for maintenance purposes:

(7) $$\begin{array}{ccc} r_{X}\left(I_{h\nu }\right) & = & r_{X,\max}\cdot \frac{I_{h\nu }}{I_{h\nu }+k_{I}}-r_{X,m}\;\;\;\;\;\mathrm{with}\;\;r_{X,\max}\\ & = & r_{h\nu ,act,\max}\cdot y_{X,h\nu } \end{array}$$

**Figure 1:**
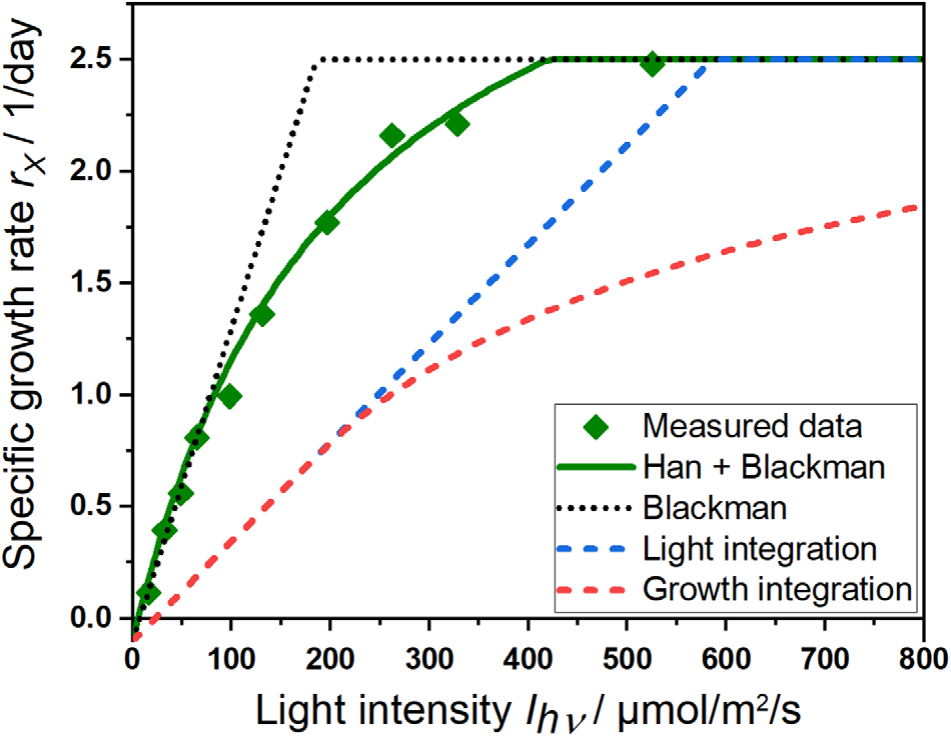
Simulations of growth kinetics. A consecutive step masks the PSU model 8 according to Blackman kinetics 6 in an ideally illuminated reactor 30 (green curve and diamonds). Ideal kinetics described by Blackman 6 assumes instant reaction and relaxation of chlorophyll (dotted black line). Other graphs encounter for steeper unidirectional light gradients as light integration (blue) and growth integration (red)

A simulation with estimated parameters is shown in Figure 1. The graphs of light and growth integration illustrate potential effects of measuring light kinetics at higher biomass concentrations as discussed in Section 2.2.

The model derived above is directly comparable to Michaelis–Menten kinetics, having the same structure of a rational function. Binding between substrate S and enzyme E according to mass action law in enzyme kinetics corresponds to light absorption of Chl. While in enzymatic systems the back reaction releases one substrate molecule, in photosynthesis the respective photon is lost. Product formation in enzyme kinetics, formulated as first order reaction of enzyme‐substrate complex to enzyme plus product, corresponds to first order transition of excited Chl to reactive Chl in the phototrophic case. In both cases mechanistic parameters are lumped to macroscopically measurable parameters (for enzymes: *r*_S,max_ = *k*_E+S→ES_ · *c*_E_; *k*_S_ = (*k*_ES→E+S_ + *k*_ES→P_)/*k*_E+S→ES_). Growth kinetics of algae can therefore often be represented by a usual rational function, but with a different physical and physiological background. Light inhibition can also be formulated considering Chl‐inactivation 19 leading to the typical quadratic term in the denominator.

Even though the state model leads to rational function, the maximum specific growth rate is often determined by a consecutive metabolic step. The impact of consecutive metabolic steps on measurable growth kinetics was firstly investigated by Blackman 6. Kinetic approaches suitable for this scheme are referred to as Blackman kinetics. Successive kinetics mask each other at different substrate concentrations or here, light intensities. In cases where two enzymatic steps are converting the same molar flux, a rational function kinetics is observed for low and middle substrate concentrations, being cut off at higher concentrations by the constant maximum turnover rate of the second step. In case the first enzymatic step is only active at low concentrations, growth can be approximated by a linear increase as the function of substrate concentration. The biological meaning is overexpression of the substrate uptake system to allow sufficient substrate uptake at low concentrations. Fitting data from a Blackman system erroneously by rational function kinetics may lead to seemingly acceptable results, but with apparent low and varying *k*_S_‐values. The linear piecewise PI‐curve (Equation 2) can be interpreted as Blackman kinetics, where light absorption corresponds to substrate uptake. Formally, Blackman kinetics leads to an additional parameter describing the maximum specific growth rate under the given conditions. The maximum specific growth rate can be caused by a consecutive internal limitation (*r*_X,max,int_) or another nutrient turnover rate *r*_X,env_ = f(*c*_Comp_) [g·g^‐1^·h^‐1^] that is stoichiometrically coupled to the one under investigation 20. The limitation of nutrient turnover is usually avoided by nutrient replete conditions during the measurement process. According to the “Law of Minimum”, the substrate concentration leading to the lowest specific growth rate determines the overall maximum specific growth rate 21.

(8) $$\begin{array}{ccc} & & r_{X,\max,true}\left(I_{h\nu }\right)\\ & & \;=\min\left(r_{X,Chl}\left(I_{h\nu }\right),\;r_{X,env}\left(c_{Comp}\right),\;r_{X,\max,\mathrm{int}}\right) \end{array}$$

**Figure 2:**
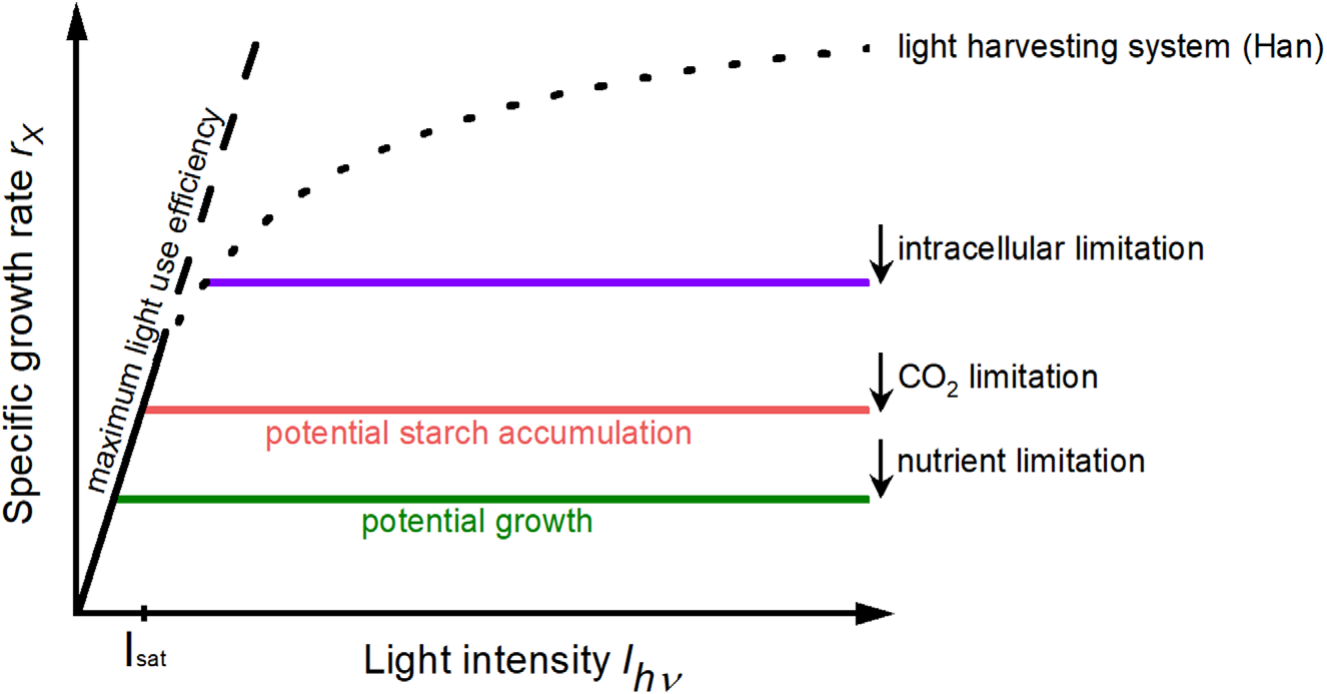
Simulation of light kinetics assuming different limiting consecutive steps. The saturation of light harvesting systems causes a hyperbolic shape of the PI‐curve. Metabolic steps following absorption confine the potential specific growth rate further. The rate of carbon fixation relates to starch production stoichiometrically, whereas the transformation of starch to active biomass requires an energy and carbon demanding respiration step

A simulation of growth kinetics assuming different limiting consecutive steps is shown in Figure 2. Many data sets in the literature can better be fit by Blackman kinetics than by pure rational function kinetics, e.g. ref. [22, 23, 24].

### 2.2 Measuring light kinetics — a view through the keyhole on physiology

PI‐curves are measured on a short‐term basis of minutes as oxygen evolution in specialized chambers 25, 26, 27, as growth in batch cultivations on a time scale of hours 28 or even including long‐term adaptation and acclimation in continuous cultivations 29. Short‐term measurements can be useful as an additional measurement during production processes to monitor the acclimation state.

Measuring light kinetics and careful quantitative interpretation can reveal further insights into the physiology of the cells under given reactor conditions. The measurement of light kinetics in short term uses low biomass concentrations in specialized chambers. Measuring the light kinetics in photobioreactors requires special modeling reactors to avoid light gradients. These reactors could be double sides illuminated plate reactors or radially illuminated tubular reactors, both at moderate biomass concentrations 30. However, recording light kinetics in the presence of higher gradients is also a necessity to follow physiological changes during relevant technical processes. Measuring the overall specific growth rate as function of incident light intensity gives only selective meaning and no predictive power.

In addition to different energetic states of chlorophyll (see Section 2.1), higher cell densities result in a smoother saturation curve due to steeper light gradients inside the photobioreactor. This gradient can be flat plate reactors approximated as an exponentially decreasing function based on the effective absorption cross section σ_X_ of the biomass. The specific growth rate then depends — assuming immediate reaction — on the position on the light path 31. Light gradients inside the reactor are not easy to measure and specific growth rates in spatial resolution are not accessible. For this reason, growth experiments are usually evaluated based on an average value for the specific growth rate *r*_X,av_ and with respect to the mean light intensity or the total absorbed photons. The absorbed photons are calculated via the incident light intensity *I*_0_ and, if necessary, the irradiation leaving the reactor on the side facing away from the light source. The mean specific photon uptake rate, usually referred to as “photon availability” [mol·g^−1^] is then determined as absorbed photons per biomass in the reactor. Describing light kinetics as the correlation of the average specific growth rate and incident light intensity or photon availability gives:

(9) $$\begin{array}{ccc} & & r_{X,av}\left(I_{h\nu ,0}\right)=r_{X}\left(\frac{1}{D_{R}}\int_{0}^{D_{R}}I_{h\nu }\left(l_{path}\right)\cdot dl_{path}\right)\\ & & \;=\frac{I_{h\nu ,0}\cdot A_{R}}{c_{X}\cdot V_{R}}=\frac{I_{h\nu ,0}}{c_{X}\cdot D_{R}}\;\;\mathrm{for}\;a\;\mathrm{plate}\;\mathrm{reactor} \end{array}$$

This approach, more correctly referred to as “light integration” 32, does not need a measurement of light gradients. But valid data can only be gained in cases, where limiting conditions apply for the whole suspension inside the reactor. Predictions in the presence of light saturation and dark parts of the reactor will lead to erroneous results.

The kinetic‐based calculation of the mean specific growth rate µ_av,I_ and comparison to measured data is not possible without any assumption concerning the light gradient inside the reactor. This more precise evaluation including light saturation is called “growth integration” as being proposed by 33, 34.

(10) $$r_{X,av,\mu }\left(I_{h\nu ,0}\right)=\frac{1}{D_{R}}\int_{0}^{D_{R}}r_{X}\left(I_{h\nu }\left(l_{path}\right)\right)\cdot dl_{path}$$

Here, the average specific growth rate is calculated by integration over local *r*_X_‐values along the light path being assumed using a given light gradient. Both approaches are compared by simulated curves in Figure 1. Growth integration, reflecting the real situation in a reactor, transforms piecewise linear kinetics as well as rational kinetics to a saturation curve. Simulation of growth integration looks similar to Monod kinetics but with a lower maximum specific growth rate and a higher limitation constant than present in the real organisms. This gives reason to assume that sometimes Monod‐type kinetics measured at high biomass concentrations is only an artifact of the transformation process. Consequently, a direct physiological interpretation of *r*_X,av_ is not admissible. To extract the real physiological kinetics, a physically reasonable approach, e.g. Han‐kinetics, has to be simulated according to Equation 10 and the unknown parameters *r*_Xmax_, *k*_I_, *r*_X,m_ have to be numerically estimated from the measured data. Investigations of the flashing light effect revealed that growth rates can be in the range between prediction by light integration and growth integration. This can be understood as the energy storage of the light harvesting complex in microalgae. However, characteristic frequencies of the flashing light effect are usually higher than the relaxation time of the harvesting pigments as predicted by the Han model.

Physiological parameters of the Han model cannot directly be extracted from measured kinetics, as they appear in linear dependencies, e.g. *n*_Chl_ and *τ*_Chl_ appear only in linear combination as n_Chl_/τ_Chl_. However, some of the physiological parameters can be measured employing optical measurement techniques, a unique feature of microalgae 35. This allows not only to check kinetic parameters for feasibility, but also gives hints for adaptation or acclimation of the cells during cultivations.

A direct measurement of the effective absorption cross section σ_X_ is possible either inline by optical sensors or offline in spectrophotometers 36, 37, 38. The measurement will help to assess the light gradient in Equation 10 and to track changing pigment contents. In addition, offline measured Chl content data (n_Chl_) delivers important information on acclimation processes and supports interpretation of *k*_I_ values. The photon yield *y*_X,hν_ is another important parameter, which can be directly estimated by measuring biomass concentration and incident light intensity and by evaluating growth integration. A value of 3 g·mol^−1^ is the theoretical maximum based on the common assumption that 10 mol photons are necessary for the fixation of 1 mol CO_2_. This will be observed only in cases, where microalgae produce high levels of starch without further conversion for growth. Transformation of starch to active biomass needs respiration for ATP generation and can reduce the photon yield up to 50%. Even lower values are obtained during lipid production as lipids have a higher heat of combustion. The lower biomass per photons does not represent a reduction of photosynthetic activity. The recalculation of photosynthetic activity needs to consider the composition of the biomass and the energy content of different constituents. Measuring light kinetics under nutrient starvation (no formation of active biomass) will allow separation of photosynthesis and anabolism. This could be another interesting means to study the photosynthetic efficiency of a given strain under given environmental conditions. Photon yield is decisive for the overall process efficiency, so it is interesting already during screening. Typical values for the parameters used in the described kinetics are given in table 2.

**Table 2:** Typical values of kinetic parameters mainly applied in the model in section 2.1

Term	Symbol	Unit	Values, Range	Comment	References
Initial slope of PI‐curve	*y*_X,I_	[(g·g^−1^·h^−1^)·(µmol·m^−2^s^−1^)^−1^]	1.7 × 10^−4^–6.0 × 10^−4^	Traditionally named α. For *r*_X_ responding to *I*_hν_.	22, 80
Photon yield	*y*_X,hν_	[g·mol^−1^]	0.2–2.1	Initial slope of PI‐curve, if normalized to absorbed photons. (recalculated from given data)	26, 64
Maximum specific growth rate	*r*_X,max_	[g·g^−1^·h^−1^]	0.02–0.15	Values are for typically applied strains; higher values for cyanobacteria.	23, 58
Light limitation constant	*k*_I_	[µmol·m^−2^s^−1^]	100–300	Higher values than this range may indicate distortion by light gradients or other limitations.	22, 80
Absorption cross section biomass	*σ*_X_	[m^2^/g]	0.1–0.3	Higher values at wavelength for Chl‐peaks, lower values, e.g. in the green gap.	36, 37, 38, 81
Relaxation time photosystem	*τ*_Chl_	[s]	10^−6^–10^−3^	Lower value as used in Figure 1, higher for explaining flashing light effect, can be measured with fast PAM.	82
Chl content in biomass	*n*_Chl_	[mg·g^−1^]	18–28	Low value for (high) light acclimated, high values for dark acclimated cells.	For other influencing parameters see: 83
Maximum specific CO_2_‐net‐uptake rate	*r*_CO2,max_	[g·g^−1^·h^−1^]	0.04–0.3	Assuming the mentioned specific growth rate range and 45 % carbon (m/m) in the formed biomass.	Calculated from *r*_X,max_
Limitation constant for CO_2_	*k*_CO2_	[%]/[µmol·L^‐1^]/[mg·L^−1^]	0.027/11/0.5	Values similar for different strains and conditions, but sensitive to pH shifts. (Solubility of CO_2_ drops with increasing temperature.)	3
Limitation constants for NO_3_^−^, NH_3_^+^, PO_4_^3−^	*k*_NO3_, *k*_NH3_, *k*_PO4_	[µmol·L^‐1^]	2–60	Highly dependent on strain, temperature, etc.	58

### 2.3 Impact of light kinetics on reactor design

Kinetic values have a direct impact on different aspects of reactor design and process development. For strain selection not only the maximum specific growth rate is decisive but also the photon yield during light limitation. Light intensity in a photobioreactor is determined by “light dilution” and “light distribution”. The so‐called light dilution is the transparent surface area of the reactor per foot print area 39, while the light distribution describes the light gradient in the suspension. For optimization, both design parameters can be calculated from kinetics. Strong sun radiation needs transformation to an average light intensity on the reactor surface slightly higher than the onset of the saturation range (e.g. for tubes or vertical panels) to achieve maximum efficiencies. At higher light intensities the first illuminated layers of the suspension are in saturation with dissipation of photons and, therefore, a loss of productivity. Light distribution along the light path is the key for calculation of the reactor thickness. The absorption cross section of the strain has to be known to perform the simulations required for reactor design. Thickness of the reactor and biomass concentration should be adapted to minimize the “dark zone” of the reactor. Only with thin reactors high biomass concentrations can be obtained. The dark fraction of the reactor provokes several disadvantages. Biomass concentrations being too high for a given reactor thickness cause a drop in productivity due to energy losses for maintenance becoming predominant in dark zones. Mixing might improve productivity by the transition of cells between different light zones. Energy is required for mixing the whole reactor volume and not only for the transition zone. In the dark parts mixing remains without a positive effect for mass transfer resulting in a waste of energy. Even seemingly simple decisions of whether a plate reactor should be oriented, N/S or E/W and the distance between two plates need a sun simulator and a kinetic based reactor model. Losses in productivity are not understandable simply by calculating reactor gauges based on photon availability.

For artificially illuminated reactors, light kinetics can give hints for calculation not only for light path but also for the light color. At first glance, red illumination seems to be favorable as absorbance shows a maximum and excess energy of photons is minimal compared to photons with shorter wavelengths. Red light is indeed appropriate for lower light intensities. Increasing light intensity and biomass concentration, while keeping the specific light availability (photons per biomass) constant, diminishes this advantage 40. Cells facing the light become saturated, absorbed but dissipated photons are missing at the side facing away from the light. A better choice at high biomass concentrations could then be yellow 41 or orange light with wavelengths at the shoulder of the red absorbance maximum to be more energy efficient and yield high productivities at medium biomass concentrations.

## 3 CO_2_ KINETICS — TAILORED SUPPLY OF CARBON SOURCE

Next to light, inorganic carbon supply is the most essential factor for microalgal cultivation. Carbon uptake on the cell level as a function of CO_2_ partial pressure in the medium is the most important kinetics to avoid carbon‐limitation and to minimize CO_2_ losses in the off‐gas.

### 3.1 Basics of CO_2_‐kinetics

The total CO_2_ demand of the cells can be calculated, as a first guess, from carbon content *e*_C,X_ of cells or its constituents. In each time interval of the cultivation the carbon dioxide transfer rate (CTR, compare Equation 12) has to be set according to this minimum requirement.

(11) $$CTR_{\min}=\frac{M_{CO2}}{M_{C}}\cdot r_{X}\cdot c_{X}\cdot e_{C,X}$$

The minimum mass of CO_2_ required for starch, here meant as the representative for the main photosynthetic product, is *Y*_CO2/X_ = *m*_CO2_/*m*_starch_ = 1.6 g·g^−1^ (starch as a unit C_6_H_10_O_5_). For maximum reduced lipids, the CO_2_ demand *Y*_CO2/Lipid_ approaches 3.1 g·g^−1^ according to mass balances. Carbon content of living microalgae cells may vary between these two key points depending on its carbohydrate, protein, or lipid content. As typical values for the carbon content of microalgae 0.5 g·g^−1^ are mentioned 42, leading to a CO_2_‐demand of *Y*_CO2/X_ = 1.8 g·g^−1^ or *r*_X_ = *y*_X,CO2_·r_CO2_ on the level of specific turnover rates. This gives an indication of the minimum carbon dioxide amount to be fed into the reactor during a cultivation.

The concentration of dissolved CO_2_ in the medium, usually expressed as partial pressure *p*_CO2_ [%saturation, e.g. 1% ≈ 409 µmol·L^−1^ ≈ 18 mg·L^−1^ at 25°C] has to be high enough to enable the cells taking up their demand. Corresponding kinetics *r*_CO2_ = *f*(*c*_CO2_) have been measured 43, 44, 45. This data can be described mostly by Michaelis–Menten‐type kinetics with a half‐saturation constant of *k*_CO2_ = 0.027% [≈11.04 µmol·L^−1^ ≈ 0.486 mg·L^−1^ at 25°C]. The knowledge of this value is basically enough to control *p*_CO2_ on an appropriate level.

A more detailed analysis reveals that data can often be better represented by Blackman kinetics introducing a second limiting step downstream of the first enzymatic step. The limiting step will cut off the slope of the rational kinetics at some point. However, this limitation step costs an additional parameter not justified by the data due to lacking parameter estimation accuracy. Parameter accuracy is limited by the persisting problem of measuring precise and stable values of *p*_CO2_ in microalgal biotechnology. Not only the accuracy is a problem of the sensors but also changing solubility, concentration of other nutrients and pH‐values. Even more, measuring HCO_3_^−^ as a potential second substrate is difficult. Recalculation based on solubility (Henry's law), dissociation constants (mass action law), and a zero‐charge balance is in principle possible, but rarely happens in practice. An overview concerning this problem is elaborated in ref. [46]. The measurement of *r*_CO2_ requires off‐gas analysis and compensation of lost CO_2_ in the effluent of continuous cultivations, e.g. by pre‐gassing of feed medium.

Carbon uptake of the cell for carbon fixation is higher than the apparent net uptake leading to an additional problem in interpretation. Algae degrade starch in respiration to obtain ATP for growth on the remaining starch fraction. At least in eukaryotic algae, the CO_2_ evolved by respiration has to be taken up again to be further used in photosynthesis. Estimations of the growth yield can consider heterotrophic growth as the benchmark, where the yield of biomass from glucose *y*_X,Gluc_ is approximately 0.4 g/g to 0.5 g/g depending on medium and cell composition 47, 48. Measured kinetics represents only the net uptake instead of the real uptake being nearly twice as high depending on the cellular composition.

### 3.2 Interpretation of measured CO_2_ kinetics values against the background of the physiology of carbon uptake

Carbon uptake as CO_2_ or HCO_3_^−^ goes along a metabolic route including (strain dependent) diffusion steps, CO_2_/HCO_3_^−^‐conversion, carbon concentration mechanisms (CCM) and, finally, the reaction with ribulose‐1,5‐bisphosphate carboxylase/oxygenase (RuBisCo) as the central carbon fixation enzyme 49.

Facing the presence of saturation kinetics in microalgae, the first diffusion step seems to not be limiting. Consequently, the focus in references is on the carbon fixation at RuBisCo. This most abundant enzyme in nature forms the main bottleneck in global carbon cycle 50. Due to its tremendous importance, much research work has been carried out to measure kinetic parameters in vivo and in vitro. Isolated RuBisCo shows in vitro typical half‐saturation constants of 740 to 1120 ppm = 0.074 to 0.112% for CO_2_ (25–38 µM 51) and maximum turnover rates of 4 s^−1^ 52. This constant is a factor 2.1 to 3.2 higher than the measured value in vivo and a factor 1.8 to 2.8 higher than the atmospheric concentration, recently 400 ppm [equilibrium in water phase 0.04% ≈ 13.6 µmol·L^−1^ ≈ 0.6 mg·L^−1^]. Biologists assume that RuBisCo has evolved in earth ages with a higher atmospheric CO_2_ concentration. Due to the “low” CO_2_ values in atmosphere nowadays, RuBisCo is expressed in high intracellular concentrations. During fast growth of microalgae the RuBisCo fractions range from 1.4 to 3.7% of the whole cell protein under nutrient replete conditions 53.

Apparent *k*_CO2_ values derived from Michaelis–Menten kinetics vary between different references and growth conditions. This is understandable as the true value of RuBisCo is masked by an additional maximum turnover rate according to Blackman kinetics. Fitting data of a process imprecisely with two consecutively active limiting steps squashed into Michaelis–Menten kinetics leads to an apparently lower half saturation constant.

Another factor is the role of oxygen partial pressure. Oxygen competes with carbon dioxide at the RuBisCo binding side. Binding oxygen leads to the so‐called “photorespiration” not to be confused with respiration in mitochondria to generate ATP for growth 54. Photorespiration is on intracellular cost of ATP and fixed CO_2_. The purpose of photorespiration is discussed as a mechanism of the cell to reduce oxygen radicals in the chloroplasts. In terms of kinetics, light respiration should be visible as reduction of photon yield and as inhibition of net carbon uptake. Only few data are available to evaluate the competition of CO_2_ and O_2_ in real technical cultivations. The measurement of *p*_O2_ in parallel is necessary but often not shown in the data sets. Oxygen “inhibition” is assumed to start above *p*_O2_ > 40%, a common value for dense and fast growing cultures 55.

Another item leading to apparently lower limitation constants for CO_2_ is CCM. CCM have the potential to shift the macroscopically visible kinetics to lower *k*_CO2_‐values compared to the values that would result from CO_2_‐measurements close to RuBisCo. Possibly, the measured k_CO2_‐values reflect the enzymatic processes in CCM more than RuBisCo itself. On the other hand, CCM leads to lower yields because of ATP expenditure. An access to verify the effect of these two mechanisms leading to a lower photon yield would be the simultaneous assessment of light and carbon uptake kinetics.

### 3.3 Consequences for gassing strategy of the photobioreactor

Optimal choice for CO_2_‐gassing is guided by the idea to keep *p*_CO2_ partial pressure in the medium at a value given by the kinetics for an anticipated *r*_X_ and to avoid CO_2_ losses in the off‐gas. In most real cases an educated guess such as *p*_CO2_ = 1% is chosen. While the whole gas stream is adjusted for sufficient mixing (e.g. 0.1 vvm), the partial pressure of *p*_CO2,Gas_ in the gas phase needs to drive the carbon dioxide transfer to the cells. The volumetric carbon dioxide transfer rate (g·L^−1^·h^−1^) over the gas–liquid interface needs to cover the volumetric carbon dioxide uptake rate (CUR [g·L^−1^·h^−1^]).

(12) $$\begin{array}{ccc} \mathrm{CTR} & = & \mathrm{CUR}=r_{CO2}\cdot c_{X}\\ & = & k_{L}a\cdot \left(c_{CO2,gas}^{*}-c_{CO2,liquid}\right) \end{array}$$

This coupled mass transfer (CTR = *f*(c_CO2,liquid_))/reaction (*r*_CO2 _= *f*(c_CO2,liquid_)) system has to always be in a dynamic equilibrium due to its short time constants.

Partial pressure in the gas phase is often controlled via pH to avoid unstable *p*_CO2_ measurements. Resulting *p*_CO2,Gas_ values are then typically in a range of 5 to 10%, what allows usage of gas from combustion processes. Assuming 10% CO_2_ in the in‐gas, 1% in the off‐gas of the bioreactor means a loss of 10% of the whole CO_2_ going into the process. pH control is also challenging as ammonia or nitrate uptake impact the pH value and pH shifts may lead to inappropriate *p*_CO2_. Again, recalculation of pH by uptake of CO_2_, HCO_3_^−^ or NH_4_^+^ is possible in principle 56, while uptake of charged compounds requires the equalization of charges between the medium and the intracellular space over the cell membrane.

Microalgae in reasonable high cell concentrations grow in the biggest part of the reactor volume below their maximum specific growth rate due to light gradients (figure 1, equ. 10). The lower specific growth rates in this part of the reactor give room to lower the p_CO2_ into the range of the measured kinetics. The lower carbon uptake for growth allows also to optimize tube lengths in tubular reactors and the gas volume fraction to reduce axial gradients. To be on the safe side, perturbation of the p_CO2_‐controller in the plant by short term CO_2_‐pulses will make possible limitations visible.

Strong aeration without any additional CO_2_ may serve as a sufficient carbon supply when the aeration rate (*k*_L_a value) is high and the light intensity inside the reactor is low due to high cell densities. The high aeration is on cost of pneumatic energy. Light and CO_2_ gradients along the bubble ascension axis also have to be considered. Excitation energy from light can be stored by the cells and used in short dark phases (ms) as known from investigations of the flashing light effect 26. Potentially, microalgae store similarly intracellular CO_2_ or HCO_3_^−^ to bridge small volume elements with low *p*_CO2_ in the reactor.

## 4 NUTRIENT UPTAKE AND STOICHIOMETRY

Media design requests the precise dosage of nutrients, above all nitrogen sources and phosphate, to enable microalgae to build up their active biomass. During continuous cultivations, e.g. in waste water treatment processes, the actual nutrient concentrations have to be high enough to prevent kinetic nutrient limitations. The apparent growth rate then results from other growth conditions than nutrient concentrations. Nitrate or ammonia and phosphate containing salts are in general limited resources and contribute to production costs. Minimization of nutrient consumption and, thus, expenditure is the overall goal. An alternative low‐priced nitrogen source is urea due to its abundance in waste water. Under alkaline conditions urea hydrolyzes to ammonia risking toxicity 57. Hydrolyzation releases, next to ammonia, CO_2_ into the medium. The CO_2_ hampers the investigation of CO_2_ kinetics. Therefore, we do not consider urea as a potential nutrient further in this review. In their natural habitat, microalgae often grow under nutrient deficiency rather than under light limitation. Consequently, studies on phytoplankton belong to the earliest published information on nutrient uptake kinetics.

### 4.1 Nutrient uptake

One example for a measured Monod‐type kinetics of a technically relevant process is given for *Dunaliella tertiolecta* at 25°C. *D. tertiolecta* shows *k*_NO3_ = 1.18 mg/L (19.1 µM) for nitrate and *k*_NH3_ *= *0.45 mg/L (25 µM) for ammonium associated with maximum specific growth rates of 1.87 day^−1^ and 1.63 day^−1^, respectively 58. In waste water processes, typically higher values are reported. Measurement inaccuracies at such low concentrations, especially under salt water conditions, or short periods of nutrient limitation cause basic difficulties to determine precise kinetics, e.g. during batch processes. In complex conditions such as waste water treatment, the stoichiometric coupling between different nutrient uptakes disables the determination of the actual limiting ion species. Ranges of typical limitation constants are given in Table 2.

Some physiological abilities of the microalgal cells regarding nutrient uptake need more attention to optimize related photo‐bioprocesses. First, many microalgae species can use ammonia and nitrate but with different preferences, usually for ammonia. In continuous cultivations this leads to lower *c*_NH3_ values than *k*_NH3_ but to higher *c*_NO3_ values than *k*_NO3_. For efficient nitrate removal, a stronger nitrogen limitation has to be adjusted. Precise measurements of such types of coupling are rare. Second, microalgae adapted to low nutrient concentrations take up different ions faster than necessary according to their macromolecular stoichiometry at a given specific growth rate. This enables the cells to store nitrogen in the form of special proteins or phosphate as polyphosphate granules. Based on these storages they grow further even at lacking nutrient supply. The storage capability makes setting up a simple *r*_X_ = *f*(*c*_PO4_) kinetics difficult. In batch cultures, the phosphate storage delays the phosphate limitation compared to the fast phosphate depletion. To determine the point of limitation, a good physiological prediction based on kinetics is necessary. The ability of microalgae to take up nutrients efficiently and to store them has brought up the idea to feed nitrogen, phosphate, and other minerals in short peaks. Nutrients will be taken up immediately and used over time, leaving only small concentrations in the medium. This is also meant to reduce bacterial contaminations.

### 4.2 Stoichiometry of macromolecular composition

Acclimation as response to light and adaptation as response to nutrient availability leads to a remarkably variable macromolecular composition of microalgae. Vice versa, macromolecular composition influences light absorbance and nutrient uptake kinetics. This is considered, e.g. in the Droop model 59, not further discussed here. One example for the mutual dependency of the cellular composition and photosynthesis is the change of Chl content with light intensity (Table 2). The Chl content influences the light gradient in the reactor and, therefore, productivity. The protein content also changes due to the stoichiometric relationship between the number of Chl‐molecules and the amount of proteins in the light harvesting antenna. Other examples are given in the following paragraphs.

The most prominent reason for stoichiometric variability of the cells is that they partially decouple anabolism from photosynthesis by the formation or usage of storage compounds. Nitrogen limitation is regularly employed to decrease anabolic activity causing accumulation of lipids and carbohydrates 60, 61 while the relative protein content decreases. Carbohydrate fractions above 60%, in exceptional cases even 70% of lipids were reported for high salinities 62. From a kinetic viewpoint we need to assess whether accumulation of storage compounds under nutrient limitation occurs on cost of cellular “stress”. Nutrient starvation may induce oxidative stress 63 due to a potentially limited turnover of essential proteins. High accumulation levels sometimes go along with a reduction of photosynthetic efficiency. Nevertheless, some strains show high intracellular storage contents without significant loss in photosynthetic efficiency 64.

Accumulation also happens under moderate nitrogen limitation but light and CO_2_ repletion as in some natural environments. After starch accumulation during the day, high decay rates (up to 30%) of the dry mass follow during the night in outdoor cultivations 65. This decrease is sometimes misinterpreted as respiration for maintenance related processes. But an increase in protein content and decrease of carbohydrates show that starch is converted to active biomass during the night. The cell simply keeps on building cell compounds on the previously accumulated starch and ongoing nitrogen uptake with the given yield *y*_X,starch_ (see Section 3), macroscopically measured as loss of dry mass. Not much kinetic information is available on this process, especially not with respect to the overall productivity, although change in composition may be important for product quality. One application example may be waste water treatment, where light is available only during the day, but nitrogen compounds have to be removed during the night as well. A kinetically based process policy could use this ability of the algal cells.

Apart from storage compounds, microalgae can also change the macromolecular composition of active biomass. In case of nitrogen repletion, all nitrogen is taken up within certain ranges. This can be proven by continuous turbidostat cultivations with increasing nitrogen source content in the feed medium. Even during constant specific growth rates, nearly all nitrogen is taken up in a certain window of operation. The nitrogen stoichiometry then leads to an increased protein content up to 0.5 g·g^−1^ as measured, e.g. by ref. [66] for *Chlorella*. Only below the nitrogen quota of 5%, a drastic reduction of the specific growth rate by the availability of nitrogen is observed. The protein fraction is then reduced to 0.3 g·g^−1^ (own data, unpublished). Such results are valuable for controlling product quality with respect to food or feed application.

**Figure 3:**
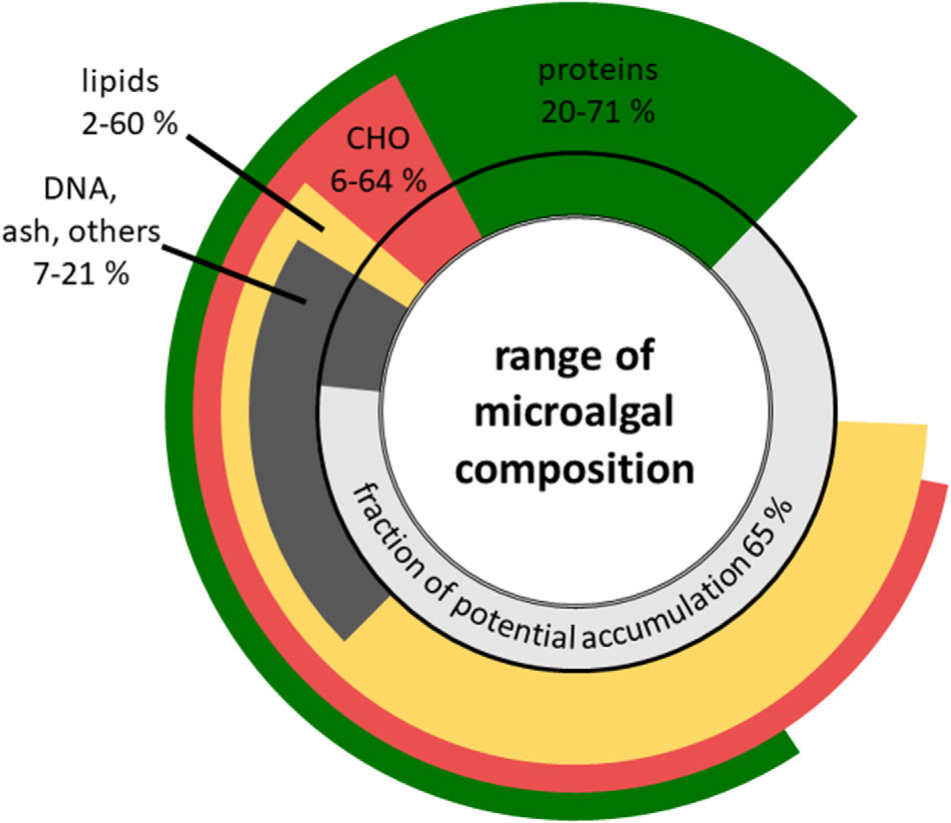
Variability of the cellular composition of microalgal dry mass in photoautotrophic cultivations. The ranges of the biomass composition are illustrated as the lower boundaries inside and the upper boundaries outside the black circle 62, 70, 71, 72, 73, 74. The lightest part of the inner circle (fraction of potential accumulation) displays the remaining fraction of the cell. This remaining fraction may theoretically be filled up with any (accumulated) biomass constituent

Ranges for cellular components such as chlorophyll, proteins, lipids, carbohydrates, nucleic acids, pigments, or ash can be found considering different process conditions and microalgae species 67. The partitioning of carbon from photosynthesis to the respective cellular components depends on the cultivation conditions 67. Next to light and CO_2_ conditions, variations of the composition are related to nutrients in terms of availability and kinetics. Temperature also plays a role in the cellular stoichiometry 68. Deviations from the optimum not only remarkably decrease growth rates, but also lead to starch production. This is discussed as a different temperature influence on photosynthesis and anabolism. The temperature aspect is not further discussed here, for review see ref. [69]. A minimum of 35% of the cell consist necessarily of certain constituents according to the reviewed data (Figure 3, 62, 70, 71, 72, 73, 74). This fraction seems to maintain the activity of essential metabolic pathways as well as the cellular structure. The remaining fraction may be composed of varying fractions of macromolecules.

Quantitative measurements of the limits of this macromolecular variability are available for singular conditions and strains. But a general assessment and deeper understanding is in demand, especially, considering possible metabolic costs visible as reduction of photosynthetic activity in production processes. Strategies to optimize the product concentration by targeted influencing carbon annotation should be based on kinetics.

## 5 CONCLUDING REMARKS

Kinetics forms the interface between cell physiology and conditions inside the reactor. The rational design of reactors, media, and processes can be based on these cell/reactor interactions. Microalgal intracellular processes connect to extracellularly measurable variables as projected by kinetics. Measuring kinetics of microalgae include specific issues such as light absorption and light gradients. Growth is a mechanistic function of the local light intensity based on the model by Han 19 and our physiological interpretation of macroscopically measurable parameters. To deduce the mean growth rate of the whole reactor, growth needs integration along the light path. Growth integration induces a deformation on measurable PI‐curves, a problem that might have hindered setting up mechanistic kinetics from measurements in the past. Numerous studies have been performed to measure light and CO_2_‐ as well as other uptake kinetics and to deduce kinetics from physiological assumptions. Recording of kinetic data has not yet been completed, but a lot of physiological knowledge is available to set up kinetics based on biological facts and mechanisms. One step of implementing mechanisms shown in this review is to distinguish between light absorption and energy usage from the absorbed photons. Combining the set of light kinetics with assumptions on carbon annotation leads to an observable macromolecular stoichiometry of the cells. However, in practice interpretation often stops on the formal level of description. Especially couplings between different kinetics such as light absorptions and CO_2_‐uptake would give hints to possible process improvement. In this review, we want to show that precise assessing of kinetics has a great potential to improve and accelerate reactor and process design. Besides given examples, other kinetics will be developed in more mechanistic precision to form a set of kinetics that copes with the complexity of the cell to a reasonable level.

## CONFLICT OF INTEREST

The authors have declared no conflict of interest. Further, the manuscript does not contain human studies or experiments using animals.
